# Supplementary material for: Facilitation of Hand Proprioceptive Processing in Paraplegic Individuals with Long-Term Wheelchair Sports Training
Source: Brain Sci. 2022 Sep 26;12(10):1295. doi: 10.3390/brainsci12101295 (PMC9599078; doi:10.3390/brainsci12101295)
Supplement: Supplementary file 1 [file brainsci-12-01295-s001.zip › brainsci-1878851-supplementary.pdf]

Supplementary Materials

Paraplegic group-specific difference between passive and active tasks

When we applied a lenient extent threshold (voxel > 150) in the evaluation of brain regions showing paraplegic group-specific differences between the passive and active tasks (paraplegic group [passive – active] – control group [passive – active]), we found clusters in the left pre-SMA and in the bilateral inferior frontal cortices, including the anterior insula, in addition to the left IPS (Figure S1A and Table S2). When we extracted parameter estimates from the 4-mm radius sphere around each peak in each participant and calculated the mean value of parameter estimates across participants for each task in each group, we found increased activity during the passive task, whereas it decreased during the active task in the paraplegic group, which was not observed in the control group (Figure S1B). This pattern of brain activity was similar to that observed in the left IPS (Figure 5B). Thus, it is likely that the left IPS worked in concert with these brain regions in the paraplegic group when performing passive and active tasks.

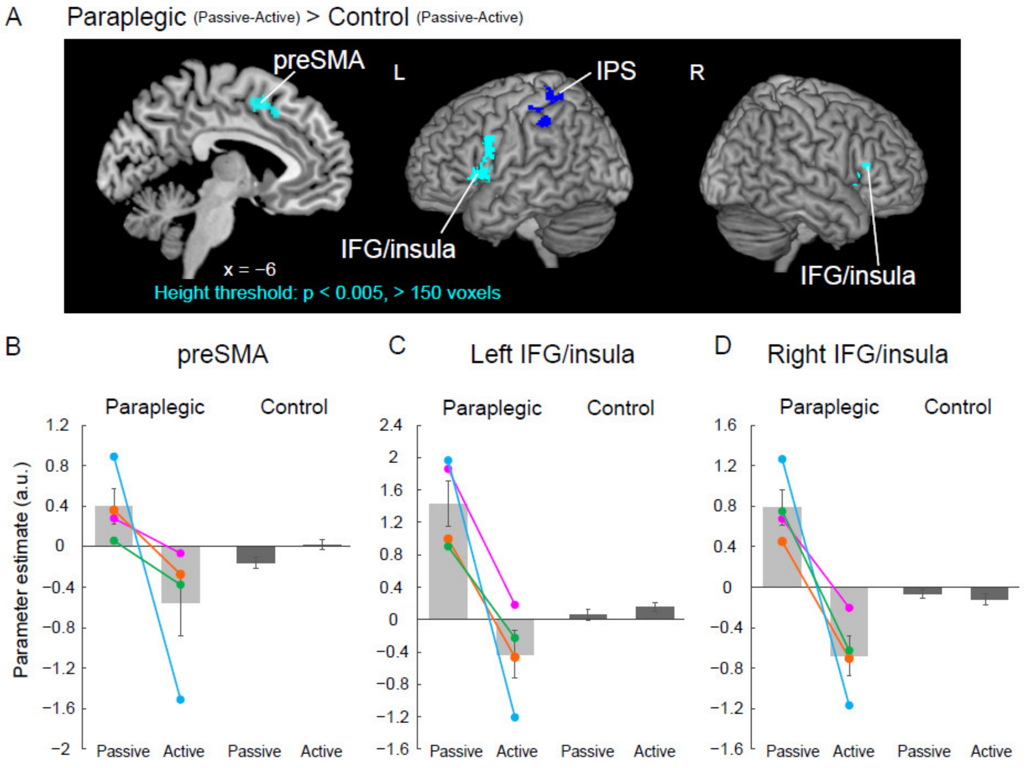

Figure S1. (**A**): Brain regions that showed significant paraplegic group-specific difference between the passive and active tasks (paraplegic group [passive – active] – control group [passive – active]). At a more lenient threshold (Height threshold, $p < 0.005$; Extent threshold, > 150 voxels), significant clusters were identified in the left pre-SMA and in the bilateral inferior frontal cortices, including the anterior insula (light blue sections), in addition to the left IPS (blue sections). (**B–D**): Bar graphs show

the mean value of brain activity (parameter estimate) from spheres with a radius of 4 mm centered on each peak of the clusters in the left pre-SMA (B), left IFG/insula (C), and right IFG/insula (D). Individual data from the paraplegic group is also plotted (pink, green, orange, and blue circles and lines represent the data obtained from paraplegic participants P1, P2, P3, and P4, respectively). Lines on the bars indicate standard errors of the mean across participants. Abbreviations: a.u., arbitrary unit; IPS, intraparietal sulcus.

Table S1. Brain regions active during passive and active tasks and those that showed task difference in the control group.

Cluster	Size (voxels)	x	y	z	T-value	Anatomical identification
**Passive task**						
L sensorimotor	3211	−34	−26	56	13.71	Area 4p
		−30	−22	64	13.19	Area 6d1
		−36	−32	62	10.40	Area 3b
L operculum	714	−42	−26	18	8.75	Area OP1
		−52	−20	38	5.67	Area 3b
R operculum/insula	323	46	2	2	6.04	Area OP4
		38	2	4	5.37	Insula
		44	2	12	4.56	Opercular cortex
R IPL	159	50	−34	24	4.69	Area PFcm
		44	−26	22	4.45	Parietal operculum
		58	−24	22	3.80	Area OP1
L thalamus	1655	−16	−20	8	4.69	Thalamus
		−26	−14	10	4.45	Putamen
		−44	2	8	3.80	Opercular cortex
R thalamus	204	14	−14	8	4.80	Thalamus
L cerebellum	291	−40	−54	−30	7.02	Cerebellum Crus I
		−30	−54	−24	6.87	Cerebellum VI
R cerebellum	2968	10	−48	−18	15.29	Cerebellum I-IV
		30	−46	−26	15.21	Cerebellum VI
		4	−62	−18	14.80	Cerebellum V (vermis)
**Active task**						
L primary motor	2809	−34	−26	54	9.75	Area 4p
		−40	−28	62	8.85	Area 4a
		−30	−22	62	8.72	Precentral gyrus

L operculum	644	−46	−28	18	11.96	Area OP1
		−54	−34	30	3.78	Supramarginal gyrus
		−60	−36	36	3.73	Area PFt
R IFG/insula	294	50	8	8	5.47	Area 44
		46	−2	6	5.39	Opercular cortex
		40	2	2	4.98	Insula
R IPL	728	48	−32	22	7.49	Area PFcm
		62	−32	38	7.02	Area PF
L putamen	1363	−26	−10	8	9.09	Putamen
		−46	0	6	7.62	Opercular cortex
		−16	−22	6	7.15	Thalamus
R putamen	168	26	−6	8	4.55	Putamen
L cerebellum	423	−30	−56	−26	9.24	Cerebellum VI
		−40	−58	−30	6.37	Cerebellum Crus I
R cerebellum	1735	20	−48	−22	10.16	Cerebellum V
		30	−44	−26	9.07	Cerebellum VI
R inferior cerebellum	425	22	−56	−52	7.01	Cerebellum VIIIb
		16	−62	−42	5.47	Dentate nucleus
**Active task − Passive task**						
Cerebellum (vermis)	625	2	−64	−22	6.61	Cerebellum Vermis VI
		10	−48	−18	5.50	Cerebellum I-IV
		4	−62	−14	4.98	Cerebellum V
L primary motor	119†	−36	−16	48	3.56	Area 4p
(M1 hand section)		−34	−24	56	3.06	Area 4p
		−38	−8	52	2.98	Precentral gyrus

Height threshold, $p < 0.005$ uncorrected; extent threshold, $p < 0.05$, FWE-corrected across the entire brain. †, an extent threshold of $p = 0.07$. For the anatomical identification of peaks, we only considered cytoarchitectonic areas available in the anatomy toolbox that had a probability greater than 30 %. The cytoarchitectonic area with the highest probability is reported for each peak. When cytoarchitectonic areas with > 30% probability were not available to determine a peak, we simply provided the anatomical location of the peak. In each cluster, we reported peaks that were more than 8 mm apart from each other in the order of larger *t*-values. R, right; L, left; IPL, inferior parietal lobule; IFG, inferior frontal gyrus.

Table S2. Brain regions that showed paraplegic group-specific difference between passive and active tasks.

Cluster	Size (voxels)	x	y	z	T-value	Anatomical identification
Paraplegic (passive − active) − control (passive − active)						
L IPS	643	−38	−34	38	5.04	Supramarginal gyrus
		−50	−36	42	4.98	Area hIP2/PFt
		−36	−44	36	4.94	Area hIP1
L IFG/insula	519	−56	12	2	5.94	Area 44
		−42	12	10	4.87	Area OP8
R IFG/insula	175	30	24	0	5.51	Area Id7
		36	30	6	4.55	IFG
		44	20	6	4.01	Area OP8
L preSMA	163	−8	8	50	5.10	Area 6mr (preSMA)
		−2	−6	58	3.88	Area 6mc (SMA)

Height threshold, $p < 0.005$ uncorrected; extent threshold, >150 voxels. For the anatomical identification of peaks, we only considered cytoarchitectonic areas available in the anatomy toolbox that had a probability greater than 30 %. The cytoarchitectonic area with the highest probability is reported for each peak. When cytoarchitectonic areas with > 30% probability were unavailable to determine a peak, we simply provided the anatomical location of the peak. In each cluster, we reported peaks that were more than 8 mm apart from each other in the order of larger *t*-values. R, right; L, left; IPS, intraparietal sulcus; IFG, inferior frontal gyrus; SMA, supplementary motor area.
